# New Drugs, Appliances, and Things Medical

**Published:** 1892-08-27

**Authors:** 


### NEW DRUGS, APPLIANCES, AND THINGS MEDICAL.

### DIAGRAMS OF THE MOUTH, FAUCES, AND LARYNX.

Messrs. J. T. Balcomb (London) have submitted to us some diagrams of the mouth, fauces, and larynx for clinical purposes. They are designed for the purpose of exactly indicating the position of parts affected in diphtheria, granular pharyngitis, laryngitis, tonsilitis, &c., &c., and in such cases are invaluable. The diagrams are in red ink, so that any pathological condition marked or sketched in black writing ink is clearly shown. They are gummed on the back, and can be readily removed and inserted in a clinical case-book. We have found them of the greatest service.

